# Early circulating tumor DNA dynamics predict fruquintinib efficacy in refractory metastatic colorectal cancer before imaging

**DOI:** 10.3389/fmed.2026.1833355

**Published:** 2026-06-12

**Authors:** Hebin Hou, Pingping Liu, Xiaohuan Dong, Wei Gai, Peng Jiang, Fan Yang, Chongli Hao

**Affiliations:** 1 Department of Gastroenterology, Tengzhou Central People's Hospital, Tengzhou, Shandong, China; 2 Department of Oncology, Tengzhou Central People's Hospital, Tengzhou, Shandong, China

**Keywords:** circulating tumor DNA, early efficacy prediction, fruquintinib, liquid biopsy, refractory metastatic colorectal cancer

## Abstract

**Background:**

Treatment options for refractory metastatic colorectal cancer remain limited, and no biomarkers predict fruquintinib benefit before imaging. This study evaluated whether early dynamics of circulating tumor DNA predict efficacy and prognosis during fruquintinib treatment.

**Methods:**

This single-center prospective cohort study enrolled patients with refractory metastatic colorectal cancer receiving fruquintinib 5 mg daily. Blood was collected at baseline, day 14, and day 28 for circulating tumor DNA testing using high-depth panel sequencing with clonal hematopoiesis filtering. Based on the day-28 maximum variant allele frequency change from baseline, patients were categorized as clearance, decrease, stable, or increase. Progression-free and overall survival were evaluated using day-28 landmark analysis. Multivariable regression models adjusted for nine clinical covariates assessed disease control rate and progression-free survival. Model performance was evaluated using Harrell's C-index, time-dependent receiver operating characteristic (ROC), bootstrap validation, and sensitivity analyses.

**Results:**

Among 120 enrolled patients, 107 completed day-28 circulating tumor DNA testing. Day-28 dynamics showed clearance in 17.8% and a decrease in 43.9%. Compared with stable/increase, the clearance and decrease groups had higher disease control rates (adjusted odds ratios, 6.92 and 3.45; both *P* < 0.01) and lower progression risk (adjusted hazard ratios, 0.41 and 0.62; both *P* < 0.05). Adding circulating tumor DNA stratification improved model discrimination for progression-free survival (ΔC-index = 0.07) and overall survival (ΔC-index = 0.06). Areas under the curve for 3-month and 6-month progression-free survival were 0.73 and 0.68. Sensitivity analyses confirmed robustness across alternative thresholds (hazard ratio range, 0.49–0.61; all *P* < 0.01). Common adverse events included hypertension (46.7%), hand-foot skin reaction (40.8%), and fatigue (36.7%), with acceptable tolerability.

**Conclusion:**

Day-28 circulating tumor DNA dynamics predict efficacy and survival during fruquintinib treatment and enhance clinical model performance. However, the modest discriminative performance (C-index 0.69) indicates that prospective validation is required before clinical implementation. These findings support further investigation of ctDNA-guided risk stratification in refractory metastatic colorectal cancer.

## 1 Introduction

Colorectal cancer remains one of the most commonly diagnosed malignancies worldwide, and for patients with metastatic disease who have exhausted multiple lines of therapy, treatment goals appropriately shift toward prolonging survival while preserving quality of life (1, 2). Despite advances in systemic therapy, the refractory metastatic colorectal cancer population, those progressing after at least two lines of standard treatment, faces a particularly poor prognosis with limited evidence-based options (3). The oral anti-angiogenic agent fruquintinib, a highly selective inhibitor of vascular endothelial growth factor receptors, has demonstrated survival benefits in this setting in the FRESCO and FRESCO-2 trials (4, 5). However, the objective response rate remains modest (4% to 6%), and substantial inter-individual heterogeneity in clinical benefit poses a persistent management dilemma (4, 6). Clinicians must navigate the tension between continuing observation on the same regimen and early strategy modification, without reliable tools to distinguish responders from non-responders before conventional imaging (3).

Current efficacy evaluation in refractory metastatic colorectal cancer relies predominantly on radiographic response per RECIST 1.1 criteria, typically performed at 8 to 12-week intervals (7). This approach carries inherent limitations: the first imaging assessment occurs weeks after treatment initiation, exposing patients with intrinsically resistant disease to potentially ineffective therapy while delaying consideration of alternative strategies (8). Serum carcinoembryonic antigen, the most accessible circulating biomarker, exhibits inconsistent kinetics due to tumor heterogeneity, treatment-related fluctuations, and the fact that approximately 30% to 40% of patients have normal levels at baseline, limiting its utility for early response prediction (9, 10).

Circulating tumor DNA has emerged as a transformative liquid biopsy tool that captures real-time tumor dynamics at the molecular level (11). Unlike static tissue genotyping, serial measurements of circulating tumor DNA can track clonal evolution, detect emerging resistance mechanisms, and quantify treatment-induced changes in tumor burden with high sensitivity (12, 13). In colorectal cancer, circulating tumor DNA (ctDNA) monitoring has been extensively validated in the adjuvant setting for detecting minimal residual disease and in the first-line setting for predicting response to chemotherapy and EGFR inhibitors (14, 15). The biological rationale for circulating tumor DNA as an early response marker rests on rapid tumor cell turnover following effective therapy: apoptosis and necrosis of treatment-sensitive clones release DNA into the circulation, while suppression of viable tumor reduces overall shedding, with changes detectable within days to weeks, well before radiographic tumor shrinkage (16, 17).

For anti-angiogenic therapies specifically, the relationship between treatment effect and circulating tumor DNA dynamics is biologically plausible yet incompletely characterized. Inhibition of vascular endothelial growth factor receptor (VEGFR) by fruquintinib induces rapid vascular pruning, perfusion remodeling, and downstream tumor cell death, thereby theoretically reducing the release of circulating tumor DNA from susceptible clones (18). Conversely, tumors employing adaptive resistance mechanisms may maintain or increase circulating tumor DNA shedding despite continued drug exposure. Early circulating tumor DNA changes could therefore serve as a pharmacodynamic readout of target engagement and a harbinger of eventual clinical outcome (19).

Despite this rationale, prospective validation of early circulating tumor DNA dynamics in later-line anti-angiogenic therapy remains sparse. Existing studies have predominantly focused on first- or second-line settings, with heterogeneous sampling schedules, variable metrics, and inconsistent threshold definitions (19, 20). More recently, ctDNA has also been explored in third-line settings, particularly in irinotecan rechallenge strategies, where it has shown promise for guiding treatment decisions. The refractory population presents unique challenges: circulating tumor DNA abundance tends to be lower; clonal hematopoiesis, particularly clonal hematopoiesis of indeterminate potential (CHIP), can generate false-positive signals if paired white blood cell sequencing is not performed; and technical factors critically influence the reliability of the results (21, 22).

The present study addresses these knowledge gaps through several innovations: fixed blood sampling at baseline, day 14, and day 28 after fruquintinib initiation; high-depth panel sequencing with unique molecular identifier-based error correction and rigorous clonal hematopoiesis filtering; a four-tiered circulating tumor DNA dynamic stratification based on percent change in maximum variant allele frequency; and evaluation of incremental predictive value when added to comprehensive clinical models incorporating established prognostic factors (23, 24). We hypothesized that early circulating tumor DNA dynamics would stratify patients into distinct risk groups before conventional imaging, with clearance or decrease indicating molecular response and translating into improved disease control and survival outcomes (25, 26). By addressing prior methodological limitations, this work aims to establish circulating tumor DNA dynamics as a clinically actionable biomarker for early risk stratification in refractory metastatic colorectal cancer.

## 2 Materials and methods

### 2.1 Study design and oversight

This single-center, prospective, observational cohort study was conducted at the Oncology Center of Tengzhou Central People's Hospital, Tengzhou, Shandong, China, between January 2022 and December 2023. The study aimed to evaluate whether early dynamic changes in circulating tumor DNA following initiation of fruquintinib predict efficacy and survival outcomes in patients with refractory metastatic colorectal cancer. Patients were enrolled consecutively at the time of treatment initiation; however, eligibility for circulating tumor DNA analysis required an adequate baseline cell-free DNA yield (≥2 ng) after laboratory processing. Baseline sample adequacy could not be determined at enrollment because cell-free DNA extraction and quantification were performed in batches after collection (one batch per week). Thus, patients with insufficient baseline cell-free DNA yield (*n* = 6) were enrolled based on the availability of a blood draw attempt but were later excluded from circulating tumor DNA analyses as soon as cell-free DNA was extracted, while remaining in the clinical outcome analyses. The protocol was approved by the Ethics Committee of Tengzhou Central People's Hospital (approval number TR20211003), and all patients provided written informed consent before enrollment. The study was conducted in accordance with the Declaration of Helsinki and Good Clinical Practice guidelines. This report follows the Strengthening the Reporting of Observational Studies in Epidemiology (STROBE) statement for cohort studies. The completed STROBE checklist is provided as Supplementary Checklist S1.

### 2.2 Study population

Eligible patients were adults aged 18 to 80 years with histologically confirmed colorectal adenocarcinoma and unresectable metastatic disease. All patients had received at least two prior lines of systemic therapy with documented disease progression and were planned to receive fruquintinib monotherapy per routine clinical practice. Fruquintinib is approved for the treatment of refractory metastatic colorectal cancer in China and represents a standard of care option at our institution. Additional inclusion criteria included Eastern Cooperative Oncology Group performance status 0–2, at least 1 measurable lesion per Response Evaluation Criteria in Solid Tumors version 1.1, and adequate hepatic, renal, and bone marrow function. Key exclusion criteria comprised prior treatment with fruquintinib, active gastrointestinal perforation or high bleeding risk, uncontrolled hypertension or severe cardiovascular disease, active hepatitis B or C with hepatic insufficiency, concomitant malignancies, and inability to provide a blood draw attempt at baseline.

### 2.3 Sample size justification

The target sample size of 120 patients was determined based on two considerations. First, to ensure stable estimation in multivariable Cox regression models with nine selected covariates, the empirical rule of 10 to 12 events per variable required approximately 90 to 108 progression events (27). Second, given the expected 90% progression rate within 12 months in this refractory population, enrollment of 120 patients was projected to yield approximately 108 events, accounting for an anticipated 10% rate of loss to follow-up or sample failure. The study was designed to provide precise effect estimates with confidence intervals (CI) rather than to test a predefined hazard ratio; therefore, all analyses are considered exploratory. The study was not registered in a clinical trial registry as it was an observational cohort study.

### 2.4 Treatment and follow-up

Patients received fruquintinib orally at a starting dose of 5 mg once daily in a 3-week-on, 1-week-off schedule (28-day cycles). Treatment continued until radiographic progression, unacceptable toxicity, investigator-determined loss of clinical benefit, or patient withdrawal. Dose modifications followed predefined rules: for grade 3 or higher treatment-related adverse events, dosing was interrupted and resumed at the next lower dose level (4 mg or 3 mg) after recovery to grade 1 or baseline (28). A maximum of two dose reductions was permitted. Permanent discontinuation was mandated for gastrointestinal perforation, arterial thrombosis, grade 3 or higher pulmonary embolism, grade 4 bleeding, persistently uncontrolled grade 3 hypertension, or any life-threatening adverse event (29). Concomitant use of strong cytochrome P450 3A4 (CYP3A4) inhibitors and inducers was prohibited. Supportive care, including antihypertensive therapy, anticoagulation when indicated, and management of dermatologic toxicity, was allowed throughout the study.

### 2.5 Study assessments and endpoints

Tumor response was assessed by computed tomography of the chest, abdomen, and pelvis, or by contrast-enhanced magnetic resonance imaging, at baseline and every 8 weeks thereafter. Two independent radiologists reviewed all images, with discrepancies adjudicated by a third senior radiologist. Response categorization followed RECIST 1.1 criteria (7).

The primary endpoint was progression-free survival, defined from the day-28 landmark to radiographic progression or death from any cause. Secondary endpoints included overall survival (from first dose to death), objective response rate (proportion achieving complete or partial response), disease control rate (proportion achieving complete response, partial response, or stable disease), and time to treatment failure (from first dose to permanent discontinuation). Circulating tumor DNA dynamics were the prespecified exposure variable for all endpoint analyses, as detailed in Section 2.7. The data cutoff date was June 30, 2025. Patients without events were censored at the last known follow-up date. Adverse events were graded according to the Common Terminology Criteria for Adverse Events version 5.0 (30).

### 2.6 Blood Sample collection and circulating tumor DNA testing

Peripheral blood samples (10 ml) were collected at three time points: baseline (within 7 days before treatment initiation), day 14 (days 12 to 16), and day 28 (days 26 to 30) of the first treatment cycle. Samples were drawn into cell-preservation tubes (Streck, Omaha, NE, USA) and processed within 2 h. After double centrifugation (first at 1,600 g for 10 min, then at 16,000 g for 10 min), plasma was aliquoted and immediately stored at −80 °C. White blood cell pellets were simultaneously separated for the purpose of filtering germline and clonal hematopoiesis variants.

Cell-free DNA was extracted from 2 to 4 ml of plasma using the QIAamp Circulating Nucleic Acid Kit (Qiagen, Hilden, Germany) according to the manufacturer's instructions. cfDNA extraction was performed in weekly batches; therefore, the time from blood collection to extraction ranged from 0 to 7 days, with a median of 3 days. This batch-extraction approach did not affect cfDNA yield or quality in our internal validation studies. Library construction employed unique molecular identifiers for error correction, followed by hybrid capture using a customized 425-gene panel covering approximately 1.2 Mb, including coding regions of genes frequently mutated in colorectal cancer and selected intron-exon junctions. The customized 425-gene panel covers full coding regions of all included genes (not limited to hotspots) and selected intron-exon junctions for fusion detection. The panel detects single-nucleotide variants, insertions, and deletions (including frameshift indels), copy number alterations, and selected gene fusions. The gene selection was based on publicly available databases of genes recurrently mutated in colorectal cancer and other solid tumors. The complete list of 425 genes with alteration types is provided in Supplementary Table S1. Sequencing was performed on the NovaSeq 6000 platform (Illumina, San Diego, CA, USA) with paired-end 150-base pair reads. The median deduplicated effective depth over target regions was 7,512 × (interquartile range, 6,123 to 9,026 × ), and the median Q30 proportion was 91.8% (interquartile range, 88.4% to 94.6%).

Sequencing data were aligned to the GRCh38 reference genome (build GRCh38.p13). Mutation calling required three or more independent mutant molecules, a deduplicated depth of at least 3,000 × at the locus, and a variant allele frequency of at least 0.1%. Copy number alterations were estimated using a fragment coverage model. Germline variants and clonal hematopoiesis were filtered by parallel sequencing of paired white blood cells; variants detected in white blood cells were annotated as hematopoietic in origin and excluded from circulating tumor DNA metrics. Sample quality control required a cell-free DNA yield of at least 2 ng, a library fragment peak between 150 and 200 base pairs, a coefficient of variation of target-region coverage uniformity of 0.25 or less, and a negative-control background mutation rate of 1 per million bases or less (31).

Samples that failed quality control were recollected once, with a new blood draw requested within 72 h of the quality control (QC) failure notification (not within 72 h of the original blood draw). The median time from initial blood draw to QC result was 5 days (interquartile range, 4 to 7 days); thus, recollection occurred approximately 5 to 8 days after the original draw, which remained within the acceptable window for ctDNA analysis based on the stability of cfDNA in cell-preservation tubes (manufacturer's specification: 7 days at room temperature). The complete workflow timeline is illustrated in Supplementary Figure S1. The replacement sample underwent the same plasma separation, storage, and batch extraction protocol as the original sample. Samples that failed again (*n* = 6 for baseline samples) were included only in clinical outcome analyses and excluded from the circulating tumor DNA dynamics set. Baseline sample adequacy could not be determined at enrollment because cell-free DNA extraction and quantification were performed in batches after collection (one batch per week). Thus, patients with insufficient baseline cell-free DNA yield (*n* = 6) were enrolled based on the availability of a blood draw attempt but were later excluded from circulating tumor DNA analyses as soon as cell-free DNA was extracted, while remaining in the clinical outcome analyses.

### 2.7 Circulating tumor DNA dynamics stratification

The primary exposure variable was the relative change in maximum variant allele frequency at day 28 compared with baseline. Maximum variant allele frequency was defined as the highest allele frequency among all non-synonymous somatic mutations detected in each sample. The maximum variant allele frequency was calculated independently at each time point as the highest allele frequency among all non-synonymous somatic mutations detected in that sample. We did not require the same mutation to carry the maximum variant allele frequency at baseline and day 28. Patients were categorized into four groups based on predefined thresholds adapted from published literature (26).

#### 2.7.1 Clearance

Baseline maximum variant allele frequency greater than 0 with all baseline mutations falling below the detection limit (0.1%) at day 28, and zero independent mutant molecules detected.

#### 2.7.2 Decrease

Reduction of at least 50% in maximum variant allele frequency relative to baseline without meeting clearance criteria.

#### 2.7.3 Stable

Change within the range of less than 50% decrease to less than 25% increase relative to baseline.

#### 2.7.4 Increase

Increase of at least 25% in maximum variant allele frequency relative to baseline or emergence of new high-confidence mutations.

Thresholds were selected based on the assay's technical coefficient of variation (15% from replicate testing) and previously published thresholds in solid tumors (8, 26). For sensitivity analyses, we also evaluated alternative thresholds (30% and 70% decreases). We used circulating tumor DNA burden (the sum of variant allele frequencies for all non-synonymous mutations) as an alternative metric. Patients with undetectable baseline circulating tumor DNA were excluded from the primary stratification but retained in the sensitivity analyses.

### 2.8 Covariates

Nine covariates were selected based on established prognostic significance in metastatic colorectal cancer (20, 23): age (< 65 years vs. ≥65 years), sex, Eastern Cooperative Oncology Group performance status (0–1 vs. 2), primary tumor laterality (left-sided vs. right-sided or transverse colon), liver metastasis (present vs. absent), number of metastatic sites (1 vs. ≥2), prior anti-vascular endothelial growth factor exposure (yes vs. no), baseline sum of longest diameters of target lesions (continuous, per 10-mm increase), and baseline maximum variant allele frequency (continuous, per 1% increase). All covariates were measured at enrollment and treated as fixed in the analyses.

The *RAS/BRAF* mutation status was collected but not included as a covariate in the primary models because it was well-balanced across ctDNA strata and was not independently associated with progression-free survival in exploratory analyses (HR 1.08, 95% CI 0.71–1.64, *P* = 0.72).

### 2.9 Statistical analysis

#### 2.9.1 Descriptive analysis

Continuous variables were summarized as medians with interquartile ranges, and categorical variables as counts with percentages. Normality was assessed using the Shapiro–Wilk test. Between-group comparisons were performed using the *t*-test or Wilcoxon rank-sum test for continuous variables and the chi-square test or Fisher's exact test for categorical variables, as appropriate.

#### 2.9.2 Landmark analysis

To avoid immortal time bias, the primary survival analysis used day 28 as the landmark time point. Only patients who were event-free and had circulating tumor DNA measured at day 28 were included in the landmark analysis set. Circulating tumor DNA dynamics were treated as a time-fixed covariate measured at the landmark. As a sensitivity analysis, we also fitted time-varying covariate Cox models that included all patients and accounted for events occurring before day 28.

#### 2.9.3 Association with disease control

Multivariable logistic regression models were constructed with disease control rate as the dependent variable, using day-28 circulating tumor DNA strata as the primary independent variable and the combined stable/increase group as the reference. Adjusted odds ratios with 95% confidence intervals were reported. Objective response rate was analyzed descriptively only due to the small number of events, and no multivariable model was fitted.

#### 2.9.4 Survival analyses

Progression-free survival and overall survival were estimated using the Kaplan–Meier method, and differences across circulating tumor DNA strata were compared using the log-rank test. Multivariable Cox proportional hazards models were fitted to estimate adjusted hazard ratios with 95% confidence intervals. The proportional hazards assumption was tested using Schoenfeld residuals. Models included the nine selected covariates and used day-28 circulating tumor DNA strata as the exposure of interest, with the stable/increase group as the reference. The Efron method was used for tie handling.

#### 2.9.5 Model performance and incremental value

Discrimination of multivariable models was assessed using Harrell's C-index. We compared models with clinical covariates alone and clinical covariates plus circulating tumor DNA stratification. The C-index was corrected for optimism using 1,000 bootstrap resamples with the Efron–Gong method. Time-dependent receiver operating characteristic curves were generated for 3 and 6-month progression-free survival, and the areas under the curves were calculated. Calibration was assessed by plotting predicted vs. observed progression probabilities at 3 and 6 months, with intercept and slope estimates from 1,000 bootstrap resamples.

#### 2.9.6 Sensitivity analyses

Four sensitivity analyses were performed to assess robustness of findings: (1) redefining “decrease” using alternative thresholds of 30% and 70% reduction in maximum variant allele frequency; (2) using circulating tumor DNA burden instead of maximum variant allele frequency as the dynamic metric; (3) excluding patients with progression or death before day 28; and (4) repeating all analyses after multiple imputation of missing covariate data.

#### 2.9.7 Missing data handling

Missing covariate data were handled using multiple imputation by chained equations with 20 imputations. Predictive mean matching was used for continuous variables, and logistic regression for binary variables. Estimates were combined using Rubin's rules. The fraction of missing information and the increase in relative variance were examined to assess imputation reliability. Circulating tumor DNA measurements at each time point and outcome variables were not imputed and were analyzed as observed.

#### 2.9.8 Statistical software and significance

All statistical analyses were performed using R version 4.3.2 (R Foundation for Statistical Computing, Vienna, Austria). All tests were two-sided with statistical significance defined as P < 0.05. Given the exploratory nature of this study, no adjustment for multiple comparisons was made; results should be interpreted accordingly.

## 3 Results

### 3.1 Patient disposition and baseline characteristics

**Figure 1:**
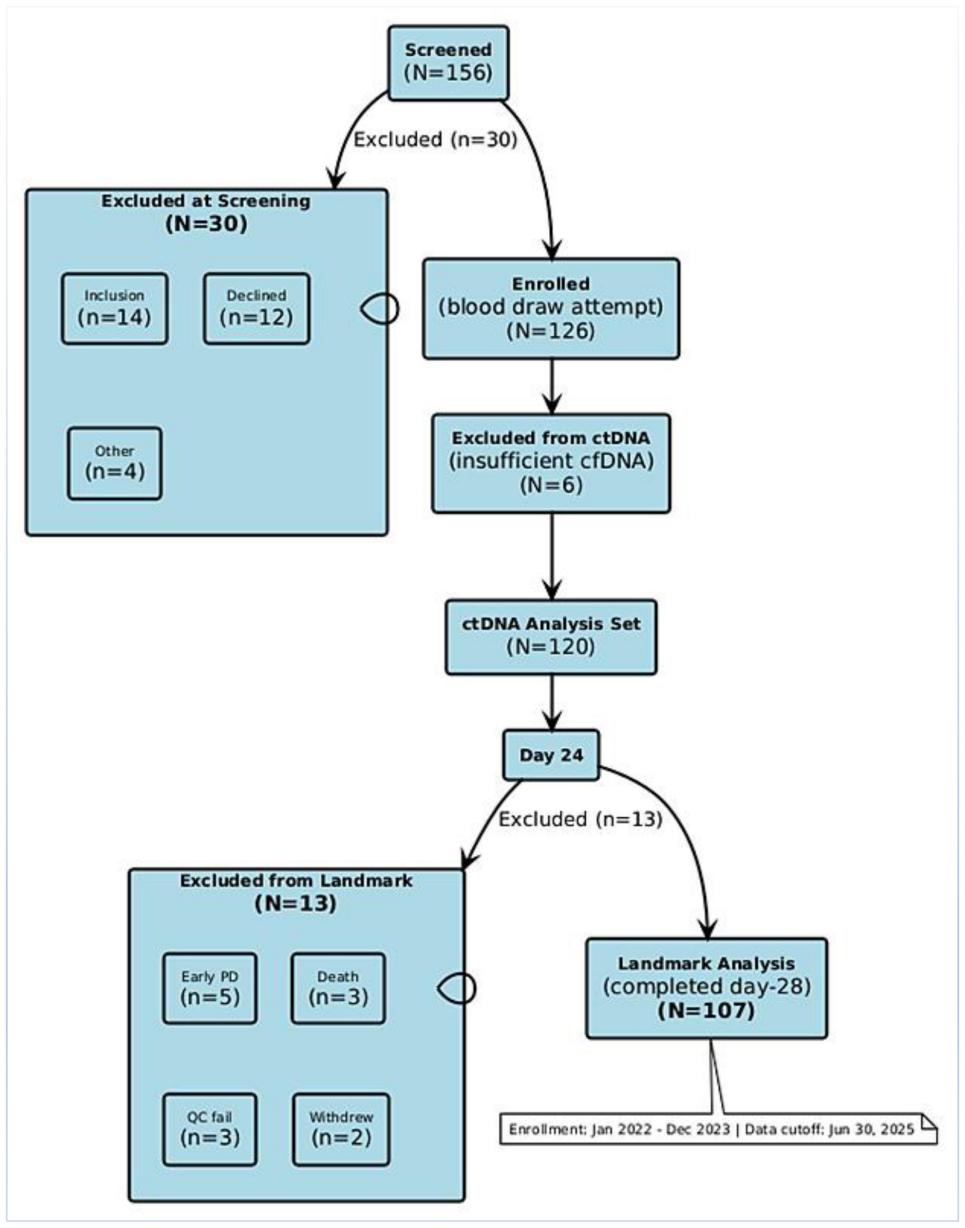
Study flow diagram. screening, enrollment, and inclusion in the day-28 landmark analysis.

Between January 2022 and December 2023, 156 patients were screened for eligibility. Of these, **30** were excluded: 14 did not meet inclusion criteria (8 had inadequate organ function, 4 had ECOG performance status >2, 2 had concomitant second malignancies), 12 declined participation, and 4 were excluded for other reasons (Figure 1). Based on a blood draw attempt, 126 patients were enrolled. After batch cfDNA extraction, 6 patients were excluded due to insufficient cfDNA yield. Thus, 120 patients had baseline circulating tumor DNA available and comprised the ctDNA analysis set. The 6 patients excluded due to insufficient baseline ctDNA did not differ significantly from the 114 patients with adequate samples in baseline characteristics or subsequent progression-free survival (Supplementary Table S2). Among these, 107 patients (89.2%) completed day-28 circulating tumor DNA testing and were included in the landmark survival analysis. The 13 patients not included in the landmark analysis comprised 5 with early disease progression before day 28, 3 who died before day 28, 3 with sample quality control failure, and 2 who withdrew consent.

Baseline demographic and clinical characteristics for the full enrolled cohort and the landmark analysis set are presented in Table 1. The median age was 59.4 years (interquartile range, 52.7 to 66.6 years), 63.3% were male, and 60.8% had left-sided primary tumors. Most patients had good performance status (ECOG 0–1, 84.2%) and extensive metastatic disease (≥2 metastatic sites, 61.7%; liver metastases, 68.3%). The median number of prior lines of systemic therapy was 2 (range, 2 to 6), with 42.5% having received three or more lines, and 61.7% had prior exposure to anti-VEGF agents. Median baseline maximum variant allele frequency was 1.87% (interquartile range, 0.42% to 6.93%), and *RAS/BRAF* mutations were present in 56.7% of patients.

**Table 1:** Baseline demographic and clinical characteristics.

Characteristic	All enrolled (*N* = 120)	Landmark analysis set (*N* = 107)
Age, years, median [IQR]	59.4 [52.7–66.6]	59.2 [52.5–66.1]
Sex, *n* (%)		
Male	76 (63.3)	66 (61.7)
Female	44 (36.7)	41 (38.3)
ECOG performance status, *n* (%)		
0–1	101 (84.2)	93 (86.9)
2	19 (15.8)	14 (13.1)
Primary tumor site, *n* (%)		
Left-sided	73 (60.8)	65 (60.7)
15.6-7.4,-14.3243pt Right-sided or transverse colon	47 (39.2)	42 (39.3)
Number of metastatic sites, *n* (%)		
1	46 (38.3)	43 (40.2)
≥2	74 (61.7)	64 (59.8)
Liver metastasis, *n* (%)		
Yes	82 (68.3)	73 (68.2)
No	38 (31.7)	34 (31.8)
Prior lines of systemic therapy, *n* (%)		
2	69 (57.5)	62 (57.9)
≥3	51 (42.5)	45 (42.1)
Prior anti-VEGF exposure, *n* (%)		
Yes	74 (61.7)	65 (60.7)
No	46 (38.3)	42 (39.3)
Baseline sum of longest diameters, mm, median [IQR]	78.6 [49.3–122.7]	76.9 [48.1–118.4]
Baseline CEA, ng/mL, median [IQR]	62.4 [6.9–428.5]	58.7 [6.3–411.2]
15.6-7.4,-14.3242ptBaseline maximum VAF, %, median [IQR]	1.87 [0.42–6.93]	1.81 [0.39–6.75]
*RAS/BRAF* mutation status, *n* (%)		
Mutant	68 (56.7)	60 (56.1)
Wild-type	52 (43.3)	47 (43.9)

IQR, interquartile range; M, median; ECOG, Eastern Cooperative Oncology Group; VEGF, vascular endothelial growth factor; CEA, carcinoembryonic antigen; VAF, variant allele frequency. Percentages may not sum to 100 due to rounding.

Patients excluded from the landmark analysis (*n* = 13) had numerically higher baseline tumor burden (median sum of longest diameters, 94.2 mm vs. 76.9 mm), higher baseline maximum variant allele frequency (median, 3.14% vs. 1.81%), and poorer performance status (ECOG 2, 30.8% vs. 13.1%) compared with those included, consistent with their early progression or death (Supplementary Table S3).

### 3.2 Circulating tumor DNA feasibility and quality control

A total of 340 plasma samples (120 baseline, 113 day 14, 107 day 28) underwent sequencing. Quality control metrics are summarized in Supplementary Table S4. The median deduplicated effective sequencing depth was 7,512 × (interquartile range, 6,123 to 9,026 × ), and the median Q30 proportion was 91.8% (interquartile range, 88.4% to 94.6%). Cell-free DNA yield was adequate across samples (median, 12.7 ng; interquartile range, 7.9 to 21.4 ng). Overall, 334 of 340 samples (98.2%) passed all predefined quality control thresholds; 6 samples (1.8%) failed and were recollected successfully within 72 h. Clonal hematopoiesis variants were detected in 17 patients (14.2%) at baseline and were successfully filtered using paired white blood cell sequencing.

### 3.3 Early circulating tumor DNA dynamics

**Figure 2:**
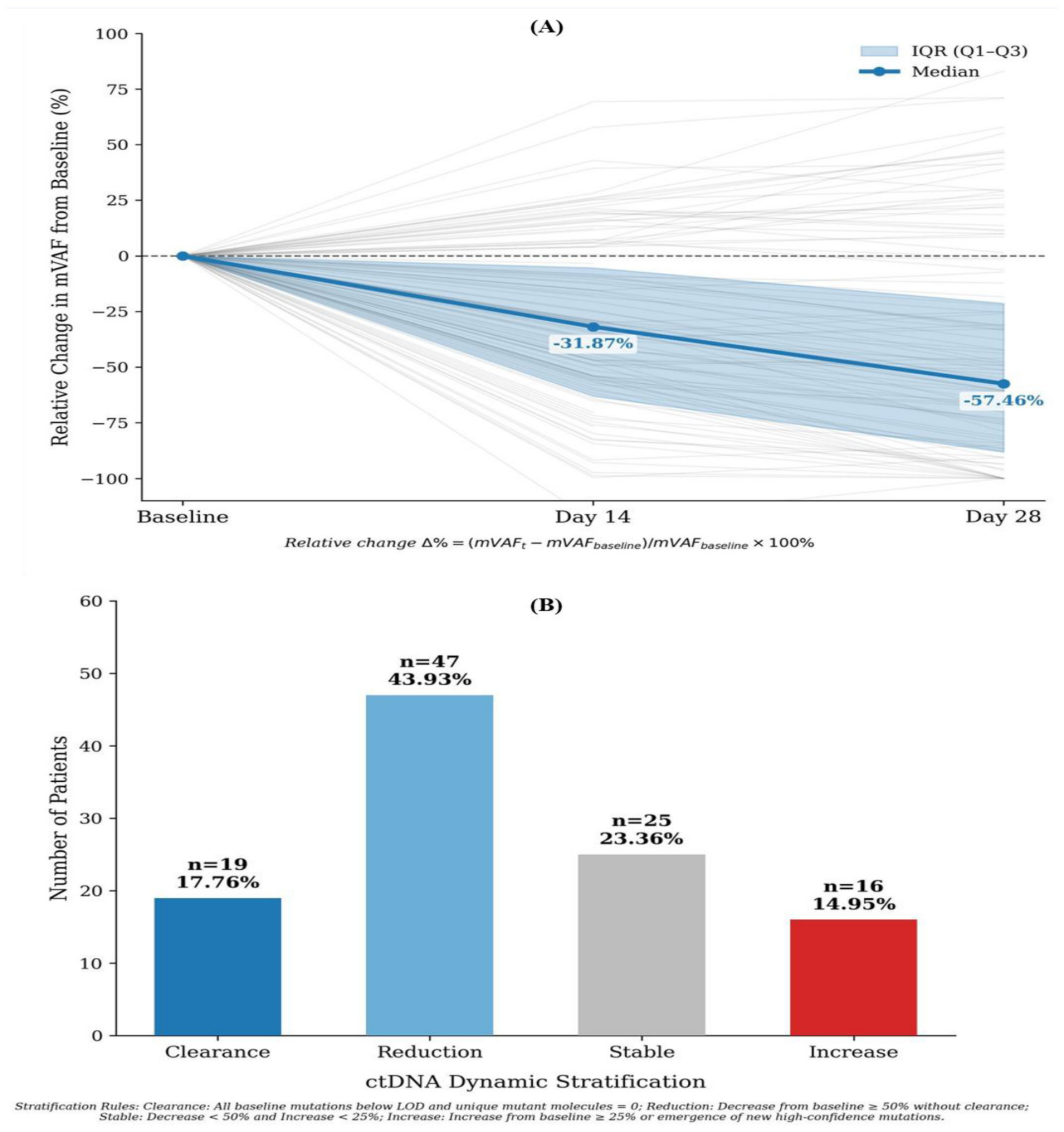
Early Circulating Tumor DNA Dynamics After Fruquintinib Initiation. **(A)** Waterfall plot showing the percent change in the maximum variant allele frequency from baseline to days 14 and 28. **(B)** Distribution of day-28 circulating tumor DNA dynamic strata (clearance, decrease, stable, increase) among 107 patients.

Among the 107 patients in the landmark analysis set, circulating tumor DNA showed a rapid downward trajectory following treatment initiation. The median relative change in maximum variant allele frequency from baseline was −31.9% (interquartile range, −58.4% to +12.7%) at day 14 and −57.5% (interquartile range, −81.2% to −16.3%) at day 28 (Figure 2A). At day 28, based on the predefined classification, 19 patients (17.8%) achieved clearance, 47 (43.9%) had a decrease, 25 (23.4%) had stable disease, and 16 (14.9%) had an increase (Figure 2B). Baseline characteristics were generally balanced across the four dynamic strata, although patients with clearance had numerically lower baseline maximum variant allele frequency and fewer liver metastases (Supplementary Table S5).

We also evaluated day-14 ctDNA dynamics as a potential earlier predictor. Using the same four-tier classification at day 14, 8 patients (7.5%) achieved clearance, 31 (29.0%) had a decrease, 42 (39.3%) had stable, and 26 (24.3%) had an increase. Day-14 ctDNA dynamics predicted progression-free survival in a landmark analysis (log-rank P = 0.01), although with lower discrimination (C-index 0.65) than day-28 (C-index 0.71). To examine the trajectory from day 14 to day 28, we constructed a concordance table (Supplementary Table S6). The overall concordance was 47.7% (51/107), with 44.9% of patients showing improvement from day-14 to day-28 and only 5.6% showing worsening. Adding day-14 dynamics to a model containing day-28 dynamics yielded minimal incremental value (ΔC-index = 0.02), confirming that day-28 dynamics capture most predictive information and serve as the optimal early landmark for risk stratification.

The observed mutation spectrum at baseline (including *TP53, APC, KRAS, NRAS, BRAF*, and *PIK3CA*) was consistent with the known genomic landscape of metastatic colorectal cancer. The complete gene list is provided in Supplementary Table S1.

### 3.4 Association with short-term efficacy

Objective responses were observed in 15 of 107 patients (14.0%), including 2 complete responses and 13 partial responses. Due to the small number of response events across strata (clearance, *n* = 7; decrease, *n* = 6; stable/increase combined, *n* = 2), the objective response rate was analyzed descriptively only and not subjected to multivariable modeling.

Disease control (complete response, partial response, or stable disease) was achieved in 65 patients (60.7%). The disease control rate differed significantly across circulating tumor DNA strata: 84.2% (16 of 19) in the clearance group, 72.3% (34 of 47) in the decrease group, and 36.6% (15 of 41) in the combined stable/increase group (*P* < 0.001 by chi-square test). In multivariable logistic regression adjusting for the nine predefined covariates, both clearance and decrease were independently associated with higher odds of disease control compared with the stable/increase reference group, as shown in Table 2. The association between day-28 ctDNA dynamics and objective response is further quantified in Supplementary Table S7.

**Table 2:** Multivariable logistic regression analysis of day-28 circulating tumor DNA dynamics and disease control rate.

ctDNA stratum	Disease control, *n*/*N* (%)	Adjusted odds ratio (95% CI)	*P* value
Clearance	16/19 (84.2)	6.92 (2.16–22.12)	0.001
Decrease	34/47 (72.3)	3.45 (1.38–8.59)	0.008
Stable/increase (reference)	15/41 (36.6)	1.00 (reference)	—

ctDNA, circulating tumor DNA; CI, confidence interval. Model adjusted for age, sex, ECOG performance status, primary tumor laterality, liver metastasis, number of metastatic sites, prior anti-VEGF exposure, baseline sum of longest diameters, and baseline maximum variant allele frequency. Stable and increasing groups were combined due to small numbers and similar disease control rates (stable, 40.0%; increase, 31.3%). Hosmer–Lemeshow goodness-of-fit *P* = 0.42.

### 3.5 Survival outcomes

Median follow-up for the landmark analysis set was 14.6 months (interquartile range, 9.8 to 20.3 months). At the data cutoff, 89 of 107 patients (83.2%) had experienced disease progression or death, and 62 patients (57.9%) had died.

**Figure 3:**
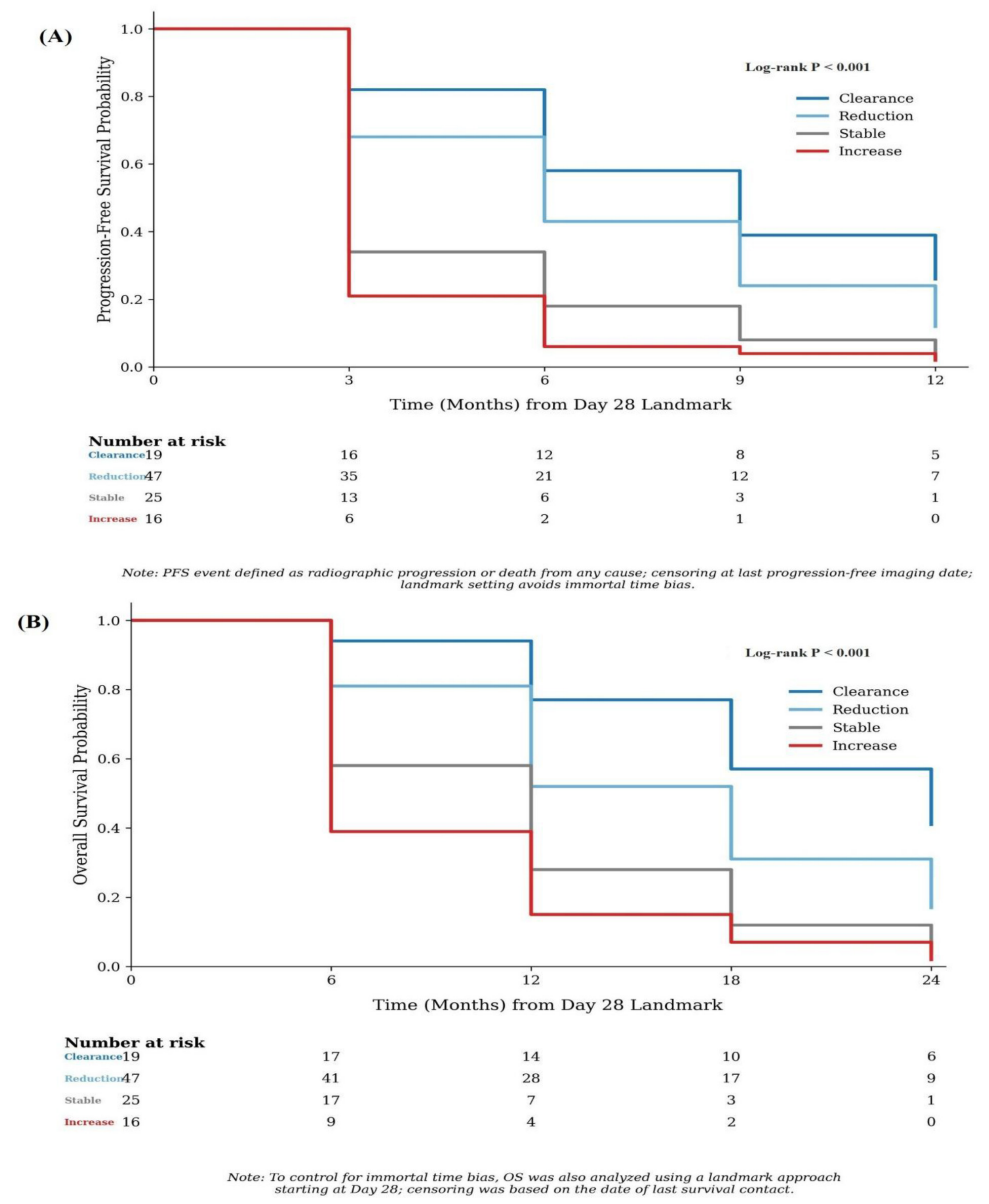
Survival outcomes according to day-28 circulating tumor DNA dynamics. **(A)** Kaplan–Meier curves for progression-free survival (log-rank *P* < 0.001). **(B)** Kaplan–Meier curves for overall survival (log-rank *P* < 0.001). Tick marks indicate censored patients.

**Figure 4:**
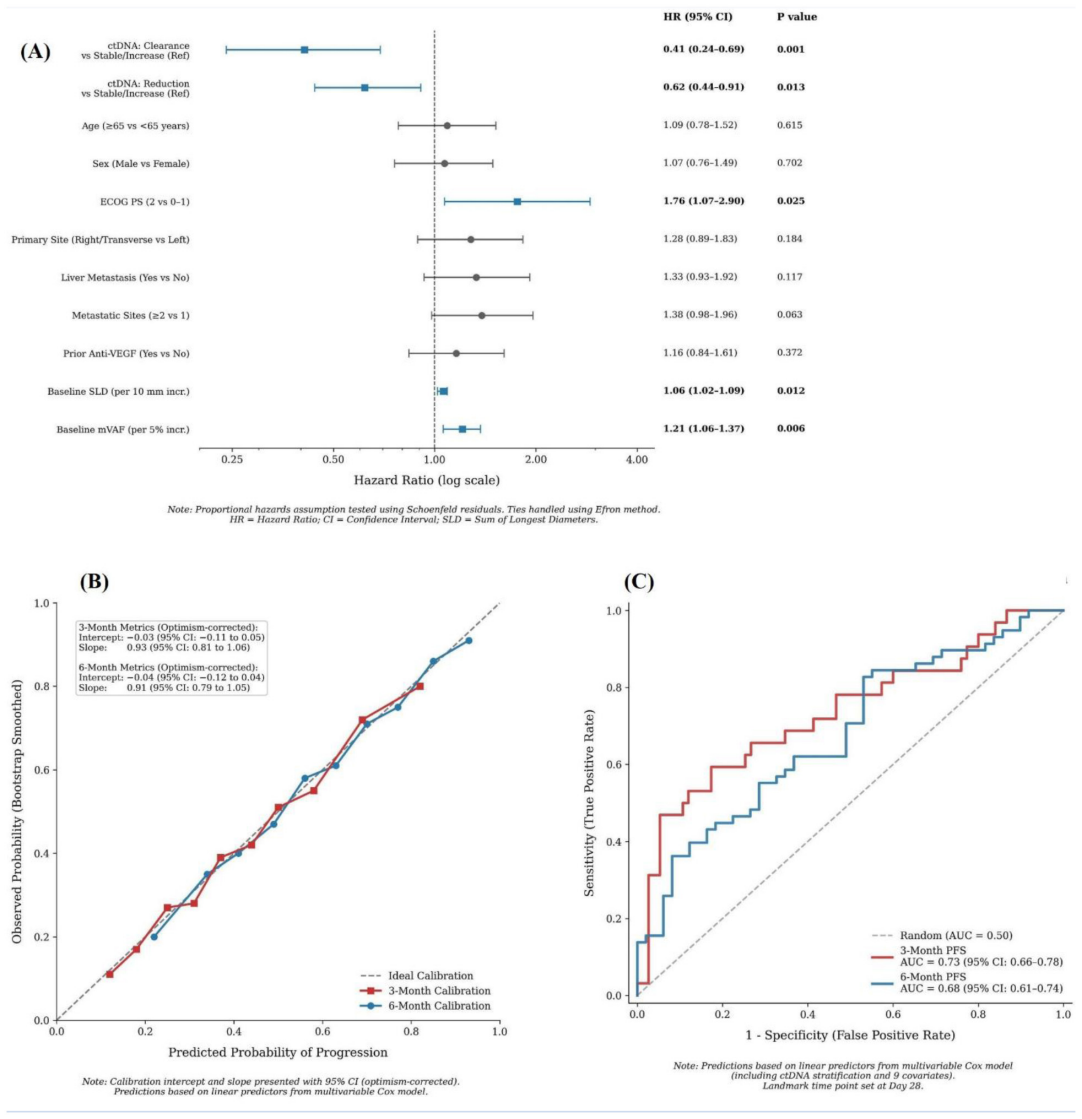
Multivariable analysis and model performance. **(A)** Forest plot of multivariable Cox regression analysis for progression-free survival. **(B)** Time-dependent receiver operating characteristic curves for prediction of 3-month and 6-month progression-free survival. **(C)** Calibration plots for predicted vs. observed progression-free survival at 3 and 6 months.

Kaplan–Meier estimates showed clear separation of progression-free survival curves according to day-28 circulating tumor DNA dynamics (log-rank *P* < 0.001; Figure 3A). Median progression-free survival was 8.2 months (95% CI, 6.4 to 10.1 months) in the clearance group, 5.4 months (95% CI, 4.3 to 6.7 months) in the decrease group, 2.9 months (95% CI, 2.1 to 3.8 months) in the stable group, and 1.9 months (95% CI, 1.4 to 2.6 months) in the increase group. Similar patterns were observed for overall survival (log-rank *P* < 0.001; Figure 3B), with median overall survival of 16.7 months (95% CI, 13.2 to 20.1 months), 12.3 months (95% CI, 10.1 to 14.8 months), 7.6 months (95% CI, 5.9 to 9.4 months), and 5.2 months (95% CI, 3.8 to 6.9 months) in the clearance, decrease, stable, and increase groups, respectively. The forest plot of the multivariable Cox regression analysis is shown in Figure 4A.

In multivariable Cox regression adjusting for all nine covariates, both clearance and decrease were independently associated with improved progression-free survival compared with the stable/increase reference group (Table 3). Other factors independently associated with worse progression-free survival included ECOG performance status 2, higher baseline sum of longest diameters, and higher baseline maximum variant allele frequency. The proportional hazards assumption was satisfied for all covariates (global Schoenfeld test, *P* = 0.23). Similar associations were observed for overall survival (Supplementary Table S8).

**Table 3:** Multivariable cox regression analysis for progression-free survival.

Variable	Adjusted hazard ratio (95% CI)	*P* value
ctDNA dynamics (vs. stable/increase)		
Clearance	0.41 (0.25–0.67)	< 0.001
Decrease	0.62 (0.41–0.93)	0.021
Age ≥65 years (vs. < 65)	1.18 (0.82–1.70)	0.37
Male sex (vs. female)	0.91 (0.64–1.29)	0.59
ECOG 2 (vs. 0–1)	1.89 (1.24–2.88)	0.003
Right-sided primary (vs. left)	1.26 (0.88–1.80)	0.20
Liver metastasis (vs. none)	1.34 (0.91–1.97)	0.14
≥2 metastatic sites (vs. 1)	1.41 (0.96–2.07)	0.08
Prior anti-VEGF exposure (vs. none)	1.19 (0.83–1.71)	0.34
Baseline SLD (per 10-mm increase)	1.12 (1.04–1.21)	0.004
Baseline maximum VAF (per 1% increase)	1.08 (1.02–1.15)	0.011

ctDNA, circulating tumor DNA; CI, confidence interval; ECOG, Eastern Cooperative Oncology Group; VEGF, vascular endothelial growth factor; SLD, sum of longest diameters; VAF, variant allele frequency. Stable and increase groups were combined as reference due to similar hazard ratios in univariable analysis (stable vs. increase: HR, 1.28; 95% CI, 0.74–2.21; *P* = 0.38).

### 3.6 Incremental predictive value of circulating tumor DNA dynamics

Adding day-28 circulating tumor DNA stratification to the clinical model (nine covariates alone) improved discrimination for both progression-free and overall survival. The Harrell's C-index for progression-free survival increased from 0.63 (95% CI, 0.57 to 0.69) with clinical covariates alone to 0.71 (95% CI, 0.66 to 0.77) after inclusion of circulating tumor DNA dynamics (ΔC = 0.07). Bootstrap-corrected values confirmed this improvement. For overall survival, the C-index increased from 0.66 (95% CI, 0.60 to 0.73) to 0.72 (95% CI, 0.66 to 0.78) (ΔC = 0.06). These findings are summarized in Table 4.

**Table 4:** Model discrimination before and after inclusion of circulating tumor DNA dynamics.

Model	Harrell's C-index apparent (95% CI)	Harrell's C-index bootstrap-corrected (95% CI)	ΔC
Progression-free survival			
Clinical model alone	0.63 (0.57–0.69)	0.62 (0.56–0.68)	—
Clinical + ctDNA dynamics	0.71 (0.66–0.77)	0.69 (0.63–0.75)	0.07
Overall survival			
Clinical model alone	0.66 (0.60–0.73)	0.64 (0.58–0.71)	—
Clinical + ctDNA dynamics	0.72 (0.66–0.78)	0.70 (0.63–0.76)	0.06

NotectDNA, circulating tumor DNA; CI, confidence interval; ΔC, change in C-index after adding ctDNA dynamics. Clinical model included age, sex, ECOG performance status, primary tumor laterality, liver metastasis, number of metastatic sites, prior anti-VEGF exposure, baseline sum of longest diameters, and baseline maximum variant allele frequency. Bootstrap correction used 1,000 resamples with the Efron–Gong optimism method.

Time-dependent receiver operating characteristic analysis demonstrated that the model incorporating circulating tumor DNA dynamics had areas under the curve of 0.73 (95% CI, 0.65 to 0.81) for predicting 3-month progression-free survival and 0.68 (95% CI, 0.60 to 0.76) for 6-month progression-free survival (Figure 4B). Calibration plots showed good agreement between predicted and observed progression probabilities at both time points, with intercepts near zero (−0.03 at 3 months, −0.04 at 6 months) and slopes near one (0.93 at 3 months, 0.91 at 6 months) after bootstrap correction (Figure 4C).

### 3.7 Sensitivity analyses

Sensitivity analyses consistently supported the main findings (Supplementary Table S9). Using alternative thresholds to define decrease (30% or 70% reduction in maximum variant allele frequency) yielded similar hazard ratios for the combined clearance/decrease group vs. stable/increase (adjusted HR range, 0.49-0.57; all *P* < 0.01). Replacing maximum variant allele frequency with circulating tumor DNA burden as the dynamic metric produced an adjusted hazard ratio of 0.61 (95% CI, 0.44 to 0.86; *P* = 0.004). Excluding patients with events before day 28 (*n* = 8) and including the remaining 111 patients in a time-varying covariate model gave an adjusted hazard ratio of 0.58 (95% CI, 0.41 to 0.84; *P* = 0.003). Multiple imputation for missing covariate data (*n* = 5 patients with incomplete baseline data) did not materially change effect estimates. The fraction of missing information ranged from 0.03 to 0.11, indicating low imputation-related uncertainty.

### 3.8 Post-progression treatment and safety

After disease progression, 91 of 120 patients (75.8%) received subsequent anticancer therapy. The most common post-progression treatments were chemotherapy, anti-VEGF agents, and regorafenib. Median time to initiation of subsequent therapy was 5.6 months (interquartile range, 4.3 to 7.9 months) for chemotherapy, 5.1 months (interquartile range, 3.9 to 7.0 months) for anti-VEGF agents, and 4.9 months (interquartile range, 3.7 to 6.6 months) for regorafenib (Supplementary Table S10).

Treatment-related adverse events of any grade occurred in 112 patients (93.3%). The most common adverse events were hypertension, hand-foot skin reaction, and fatigue. Grade 3 or 4 adverse events occurred in 48 patients (40.0%), with hypertension being the most frequent grade 3–4 event. Dose reductions due to adverse events were required in 28 patients (23.3%), and treatment discontinuation due to adverse events occurred in 9 patients (7.5%). No treatment-related deaths occurred. Detailed safety data are presented in Table 5.

**Table 5:** Treatment-related adverse events.

Adverse event	Any grade, *n* (%)	Grade 3–4, *n* (%)	Leading to dose reduction, *n* (%)	Leading to discontinuation, *n* (%)
Hypertension	56 (46.7)	22 (18.3)	17 (14.2)	3 (2.5)
Hand-foot skin reaction	49 (40.8)	13 (10.8)	11 (9.2)	2 (1.7)
Fatigue	44 (36.7)	8 (6.7)	5 (4.2)	1 (0.8)
Decreased appetite	41 (34.2)	5 (4.2)	3 (2.5)	1 (0.8)
Proteinuria	38 (31.7)	9 (7.5)	7 (5.8)	1 (0.8)
Diarrhea	33 (27.5)	7 (5.8)	4 (3.3)	1 (0.8)
Hypothyroidism	23 (19.2)	0 (0)	2 (1.7)	0 (0)
ALT increased	19 (15.8)	3 (2.5)	2 (1.7)	0 (0)
AST increased	17 (14.2)	2 (1.7)	1 (0.8)	0 (0)
Bleeding events	14 (11.7)	4 (3.3)	2 (1.7)	1 (0.8)
Venous thrombosis/PE	7 (5.8)	4 (3.3)	1 (0.8)	2 (1.7)
Gastrointestinal perforation	2 (1.7)	2 (1.7)	0 (0)	2 (1.7)
Hypertension	56 (46.7)	22 (18.3)	17 (14.2)	3 (2.5)
Hand-foot skin reaction	49 (40.8)	13 (10.8)	11 (9.2)	2 (1.7)
Fatigue	44 (36.7)	8 (6.7)	5 (4.2)	1 (0.8)
Decreased appetite	41 (34.2)	5 (4.2)	3 (2.5)	1 (0.8)
Proteinuria	38 (31.7)	9 (7.5)	7 (5.8)	1 (0.8)
Diarrhea	33 (27.5)	7 (5.8)	4 (3.3)	1 (0.8)
Hypothyroidism	23 (19.2)	0 (0)	2 (1.7)	0 (0)
ALT increased	19 (15.8)	3 (2.5)	2 (1.7)	0 (0)
AST increased	17 (14.2)	2 (1.7)	1 (0.8)	0 (0)
Bleeding events	14 (11.7)	4 (3.3)	2 (1.7)	1 (0.8)
Venous thrombosis/PE	7 (5.8)	4 (3.3)	1 (0.8)	2 (1.7)
Gastrointestinal perforation	2 (1.7)	2 (1.7)	0 (0)	2 (1.7)

## 4 Discussion

This prospective cohort study demonstrates that early circulating tumor DNA dynamics predict efficacy and survival outcomes in patients with refractory metastatic colorectal cancer receiving fruquintinib. Using fixed-sampling at day 28 after treatment initiation, with rigorous clonal hematopoiesis filtering, we found that patients achieving clearance or a decrease in circulating tumor DNA had significantly higher disease control rates and longer progression-free and overall survival than those with stable or increasing levels of circulating tumor DNA. These associations remained significant after adjustment for nine predefined clinical covariates, and incorporating circulating tumor DNA dynamics improved model discrimination beyond clinical factors alone. The findings were robust across multiple sensitivity analyses, including alternative thresholds and dynamic metrics.

The biological rationale for these observations is well established. Circulating tumor DNA originates from apoptotic and necrotic tumor cells as well as active release, providing a real-time measure of tumor burden and clonal activity (13). Following vascular endothelial growth factor receptor inhibition by fruquintinib, suppression of angiogenesis and vascular remodeling rapidly affects tumor cell turnover, reducing circulating tumor DNA shedding from sensitive clones (16). Conversely, stable or increasing levels may indicate primary resistance via mechanisms such as hypoxia-driven clonal selection, vascular co-option, or activation of alternative angiogenic pathways (18). Our observation that ctDNA changes at day 28, well before the first scheduled imaging at week 8, stratifies patients into distinct prognostic groups and aligns with this biological framework.

Our findings extend prior work on circulating tumor DNA as an early response biomarker in colorectal cancer. Parikh et al. (8) demonstrated that serial monitoring of circulating tumor DNA predicted response to systemic therapy in metastatic gastrointestinal cancers, with changes detectable as early as 4 weeks after treatment initiation. Similarly, Amatu et al. (17) reported that high circulating methylated DNA predicted poor outcomes in patients receiving regorafenib, another later-line anti-angiogenic agent. However, those studies employed heterogeneous sampling schedules and did not systematically address clonal hematopoiesis filtering. The present study advances the field by implementing fixed time points aligned with the first treatment cycle, rigorous, unique molecular identifier-based error correction, and paired white blood cell sequencing to eliminate clonal hematopoiesis-related false signals (21, 22).

The incremental value of circulating tumor DNA dynamics beyond established clinical prognostic factors deserves emphasis. Models incorporating only clinical covariates, including ECOG performance status, tumor burden, and prior treatment exposure, demonstrated modest discrimination (C-index 0.63 for progression-free survival), consistent with previous reports in metastatic colorectal cancer (23). Adding day-28 circulating tumor DNA dynamics improved the C-index to 0.71, representing a clinically meaningful enhancement. This suggests that molecular response captures treatment-specific information not reflected in baseline clinical characteristics, supporting the integration of early circulating tumor DNA assessment into routine risk stratification.

Our results also inform ongoing efforts to define response criteria for liquid biopsy biomarkers. The four-tiered classification system, clearance, decrease, stable, and increase, provided clear prognostic separation, with median progression-free survival ranging from 8.2 months in the clearance group to 1.9 months in the increase group. The stability of findings across alternative thresholds (30% and 70% decrease) suggests that the specific cutpoint is less critical than the overall directional signal, consistent with observations from Weber et al. (26) in non-small cell lung cancer. This flexibility may facilitate clinical implementation, as laboratories can adapt thresholds to their specific assay characteristics while maintaining prognostic utility.

Our four-tier classification aligns qualitatively with the recently proposed Liquid Biopsy Response Evaluation Criteria in Solid Tumors (LB-RECIST) framework (6). Specifically, our ‘clearance' corresponds to LB-RECIST molecular clearance, ‘decrease' (≥50% reduction) corresponds to molecular response, ‘stable' corresponds to molecular stable disease, and ‘increase' (≥25% increase) corresponds to molecular progression. However, we acknowledge that LB-RECIST uses aggregate VAF (aggVAF) with ±10% thresholds (6), while our quantitative thresholds (50% decrease, 25% increase) are adapted from Weber et al. (26) in non-small cell lung cancer. Our mVAF-based classification is therefore qualitatively aligned with LB-RECIST categories but differs in the specific metric and threshold magnitudes. This distinction should be considered when comparing across studies. To our knowledge, this is one of the first prospective studies to validate LB-RECIST criteria in this setting.

Several interventional trials have already demonstrated the feasibility of ctDNA-guided treatment strategies in metastatic colorectal cancer. As comprehensively reviewed by Roazzi et al. (32), trials such as CRICKET (33) and CHRONOS have shown that ctDNA monitoring can identify patients likely to benefit from *anti-EGFR* rechallenge in *RAS/BRAF* wild-type patients. Similarly, ctDNA-guided treatment-switch paradigms have been explored in the adjuvant setting (DYNAMIC trial) (14) and are currently being evaluated in metastatic disease. However, to our knowledge, no interventional trial has yet tested ctDNA-guided treatment decisions for later-line anti-angiogenic agents such as fruquintinib. Our study, therefore, provides a foundational rationale for such trials, demonstrating that early ctDNA dynamics predict outcomes and could guide early treatment switches or continued therapy.

The safety profile observed in our cohort aligned with that observed in previous fruquintinib trials (4, 28). Hypertension, hand-foot skin reaction, and fatigue were the most common adverse events, with grade 3–4 events occurring in 40% of patients and treatment discontinuation in 7.5%. Importantly, no new safety signals emerged, confirming the tolerability of fruquintinib in this heavily pretreated population.

The objective response rate observed in our cohort (14.0%) was higher than the 4%−6% reported in the phase 3 FRESCO and FRESCO-2 trials (4, 5). This difference may be explained by several factors. First, our study enrolled patients with detectable baseline ctDNA, which may select for a biologically distinct population with different tumor characteristics. Second, real-world cohorts often have less stringent eligibility criteria than randomized trials, potentially leading to different response rates. Third, the smaller sample size (*n* = 107) increases the potential for random variation. Nevertheless, our ORR remains within the range reported in other real-world studies of fruquintinib.

### 4.1 Limitations and future directions

Several limitations warrant consideration. (1) Single-center, modest sample size; excluded patients had higher baseline tumor burden and ctDNA levels, potentially introducing selection bias. External validation in larger, multicenter cohorts is essential. (2) This observational cohort study was not registered in a clinical trial registry, as registration was not required by our institution. The complete study protocol (sample size, ctDNA stratification, thresholds, endpoints) is provided as Supplementary Protocol S1, timestamped before enrollment. (3) Observational design cannot exclude unmeasured confounding; we did not adjust for cumulative drug exposure or adherence. (4) A small number of objective response events limited the assessment of radiographic response associations. (5) Heterogeneous post-progression treatments could affect overall survival; however, subsequent therapy patterns were similar across ctDNA strata, suggesting this is unlikely to account for observed survival differences. (6) ctDNA thresholds, informed by assay variability and literature, require prospective validation. (7) The 425-gene panel and sequencing workflow may not be directly generalizable to other platforms. (8) MSI/MMR status was not collected. dMMR tumors are rare in refractory mCRC, and their inclusion is unlikely to bias overall estimates. Fruquintinib targets angiogenesis, not immune checkpoints, and there is no evidence that ctDNA dynamics differ by MMR status. Nevertheless, future studies should include MMR status as a covariate. Day-14 ctDNA analyses showed lower discrimination (C-index 0.65 vs. 0.71), with only 47.7% concordance between day-14 and day-28, suggesting that 2 weeks may be insufficient for detectable response in many patients (9). Tissue-plasma concordance data unavailable (plasma-only design). However, rigorous quality control measures were implemented: high-depth sequencing (7,512 × ), UMI error correction, paired WBC sequencing for CHIP filtering, and VAF ≥0.1% with ≥3 mutant molecules, consistent with best practices (34). (10) Our mVAF approach does not require tracking the same mutation across time points. In theory, subclonal replacement could conflate true burden reduction with clonal shift. However, a sensitivity analysis using ctDNA burden (robust to clonal shifts) yielded consistent results (HR 0.61, *P* = 0.004), suggesting minimal bias. (11) The absolute C-index of 0.69 represents modest discriminative performance. If the ctDNA increase guided early treatment switch, 16% of patients would be considered; among these, 81.3% had PD as best response, while 18.7% had SD and would have been inappropriately switched. However, this estimate is based on only 3 patients (18.7% of 16) and should be interpreted with caution. Patients with ctDNA stable (23.4% of the landmark set, DCR 40%) would also be candidates for early treatment modification in a clinically plausible algorithm. Thus, prospective validation in larger cohorts is required before clinical implementation.

Future research should focus on several directions. Prospective multicenter validation studies with predefined circulating tumor DNA-driven treatment algorithms are needed to determine whether early molecular response can guide clinical decisions, such as continuing fruquintinib in patients with clearance or decrease vs. switching to alternative therapies in those with stable or increasing levels. Integration with emerging liquid biopsy technologies, including methylation-based circulating tumor DNA detection, may further enhance sensitivity and specificity (25). Additionally, exploring the genomic alterations underlying primary resistance, through analysis of baseline circulating tumor DNA characteristics and emergent mutations in patients with increasing levels, could identify novel therapeutic targets (20). Finally, health economic analyses should evaluate the cost-effectiveness of routine early circulating tumor DNA monitoring in this refractory population.

## 5 Conclusion

In patients with refractory metastatic colorectal cancer treated with fruquintinib, early circulating tumor DNA dynamics at day 28 predict disease control, progression-free survival, and overall survival independent of established clinical prognostic factors. Incorporating circulating tumor DNA stratification improves the performance of clinical prediction models and enables the identification of patients with divergent outcomes before the first imaging assessment. However, the modest discriminative performance (C-index 0.69) and risk of misclassification (approximately 1 in 5 patients) indicate that prospective validation in interventional trials is required before clinical implementation. These findings provide a foundation for future studies investigating ctDNA-guided treatment decisions in later-line therapy.

## Data Availability

The original contributions presented in the study are included in the article/Supplementary material, further inquiries can be directed to the corresponding author.

## References

[1] Feria A Times M. Effectiveness of standard treatment for stage 4 colorectal cancer: traditional management with surgery, radiation, and chemotherapy. Clin Colon Rectal Surg. (2023) 37:62–5. doi: 10.1055/s-0043-1761420 38322607 PMC10843885

[2] Cremolini C Chalabi M Elez E Fassan M Gelli M Goéré D . Metastatic colorectal cancer: ESMO clinical practice guideline for diagnosis, treatment and follow-up. Ann Oncol. (2023) 34:10–32. doi: 10.1016/j.annonc.2022.10.003 41990853

[3] Fernández-Montes A Grávalos C Pericay C Safont MJ Benavides M Élez E . Current options for third-line and beyond treatment of metastatic colorectal cancer. Spanish TTD group expert opinion. Clin Colorectal Cancer. (2020) 19:165–77. doi: 10.1016/j.clcc.2020.04.003 32507561

[4] Dasari A Lonardi S Garcia-Carbonero R Elez E Yoshino T Sobrero A . Fruquintinib versus placebo in patients with refractory metastatic colorectal cancer (FRESCO-2): an international, multicentre, randomised, double-blind, phase 3 study. Lancet. (2023) 402:41–53. doi: 10.1016/S0140-6736(23)00772-9 37331369

[5] Li J Qin S Xu RH Shen L Xu J Bai Y . Effect of Fruquintinib vs Placebo on overall survival in patients with previously treated metastatic colorectal cancer: the FRESCO randomized clinical trial. JAMA. (2018) 319:2486–96. doi: 10.1001/jama.2018.7855 29946728 PMC6583690

[6] Gouda MA Janku F Wahida A Buschhorn L Schneeweiss A Abdel Karim N . Liquid biopsy response evaluation criteria in solid tumors (LB-RECIST). Ann Oncol. (2024) 35:267–75. doi: 10.1016/j.annonc.2023.12.007 38145866

[7] Eisenhauer EA Therasse P Bogaerts J Schwartz LH Sargent D Ford R . New response evaluation criteria in solid tumours: revised RECIST guideline (version 11). Eur J Cancer. (2009) 45:228–47. doi: 10.1016/j.ejca.2008.10.026 19097774

[8] Parikh AR Mojtahed A Schneider JL Kanter K Van SEE Fetter IJ . Serial ctDNA monitoring to predict response to systemic therapy in metastatic gastrointestinal cancers. Clin Cancer Res. (2020) 26:1877–85. doi: 10.1158/1078-0432.CCR-19-3467 31941831 PMC7165022

[9] Hall C Clarke L Pal A Buchwald P Eglinton T Wakeman C . A review of the role of carcinoembryonic antigen in clinical practice. Ann Coloproctol. (2019) 35:294–305. doi: 10.3393/ac.2019.11.13 31937069 PMC6968721

[10] Nicholson BD Shinkins B Pathiraja I Roberts NW James TJ Mallett S . Blood CEA levels for detecting recurrent colorectal cancer. Cochrane Database Syst Rev. (2015) 12:CD011134. doi: 10.1002/14651858.CD011134.pub2 26661580 PMC7092609

[11] Krell M Llera B Brown ZJ. Circulating tumor DNA and management of colorectal cancer. Cancers. (2023) 16:21. doi: 10.3390/cancers16010021 38201448 PMC10778183

[12] Malla M Loree JM Kasi PM Parikh AR. Using circulating tumor DNA in colorectal cancer: current and evolving practices. J Clin Oncol. (2022) 40:2846–57. doi: 10.1200/JCO.21.02615 35839443 PMC9390824

[13] Stejskal P Goodarzi H Srovnal J Hajdúch M van 't Veer LJ Magbanua MJM. Circulating tumor nucleic acids: biology, release mechanisms, and clinical relevance. Mol Cancer. (2023) 22:15. doi: 10.1186/s12943-022-01710-w 36681803 PMC9862574

[14] Tie J Cohen JD Lahouel K Lo SN Wang Y Kosmider S . Circulating tumor DNA analysis guiding adjuvant therapy in stage II colon cancer. N Engl J Med. (2022) 386:2261–72. doi: 10.1056/NEJMoa2200075 35657320 PMC9701133

[15] Chen G Peng J Xiao Q Wu H Wu X Wang F . Postoperative circulating tumor DNA as markers of recurrence risk in stages II to III colorectal cancer. J Hematol Oncol. (2021) 14:80. doi: 10.1186/s13045-021-01089-z 34001194 PMC8130394

[16] Zhao S Wang W Li J Li Z Liu Z Zhang S . Clinical research progress of fruquintinib in the treatment of malignant tumors. Invest New Drugs. (2024) 42:612–22. doi: 10.1007/s10637-024-01476-6 39352649 PMC11625063

[17] Amatu A Schirripa M Tosi F Lonardi S Bencardino K Bonazzina E . High circulating methylated DNA is a negative predictive and prognostic marker in metastatic colorectal cancer patients treated with Regorafenib. Front Oncol. (2019) 9:622. doi: 10.3389/fonc.2019.00622 31355139 PMC6640154

[18] Haibe Y Kreidieh M El HH Khalifeh I Mukherji D Temraz S . Resistance mechanisms to anti-angiogenic therapies in cancer. Front Oncol. (2020) 10:221. doi: 10.3389/fonc.2020.00221 32175278 PMC7056882

[19] Vidal J Muinelo L Dalmases A Jones F Edelstein D Iglesias M . Plasma ctDNA RAS mutation analysis for the diagnosis and treatment monitoring of metastatic colorectal cancer patients. Ann Oncol. (2017) 28:1325–32. doi: 10.1093/annonc/mdx125 28419195 PMC5834035

[20] Manca P Corallo S Lonardi S Fucà G Busico A Leone AG . Variant allele frequency in baseline circulating tumour DNA to measure tumour burden and to stratify outcomes in patients with RAS wild-type metastatic colorectal cancer: a translational objective of the Valentino study. Br J Cancer. (2022) 126:449–55. doi: 10.1038/s41416-021-01591-8 34811502 PMC8810873

[21] Croitoru VM Cazacu IM Popescu I Paul D Dima SO Croitoru AE . Clonal Hematopoiesis and Liquid Biopsy in Gastrointestinal Cancers. Front Med. (2022) 8:772166. doi: 10.3389/fmed.2021.772166 35127745 PMC8814311

[22] Chan HT Chin YM Nakamura Y Low SK. Clonal hematopoiesis in liquid biopsy: from biological noise to valuable clinical implications. Cancers. (2020) 12:2277. doi: 10.3390/cancers12082277 32823942 PMC7463455

[23] Procaccio L Bergamo F Gatti M Chiusole B Tierno G Bergo E . The oncological multidimensional prognostic index is a promising decision-making tool: A real-world analysis in older patients with metastatic colorectal cancer. Eur J Cancer. (2022) 177:112–9. doi: 10.1016/j.ejca.2022.09.023 36335781

[24] Siena S Sartore-Bianchi A Garcia-Carbonero R Karthaus M Smith D Tabernero J . Dynamic molecular analysis and clinical correlates of tumor evolution within a phase II trial of panitumumab-based therapy in metastatic colorectal cancer. Ann Oncol. (2018) 29:119–26. doi: 10.1093/annonc/mdx504 28945848 PMC5834114

[25] Raunkilde L Hansen TF Andersen RF Havelund BM Thomsen CB Jensen LH. Gene methylation in circulating tumor DNA as an early biomarker for treatment effect in metastatic colorectal cancer. Cancers. (2022) 14:4459. doi: 10.3390/cancers14184459 36139621 PMC9496936

[26] Weber S van dLP Donker HC Schlange T Timens W Tamminga M . Dynamic changes of circulating tumor DNA predict clinical outcome in patients with advanced non-small-cell lung cancer treated with immune checkpoint inhibitors. JCO Precis Oncol. (2021) 5:1540–53. doi: 10.1200/PO.21.00182 34994642

[27] Harrell FE. Regression Modeling Strategies: With Applications to Linear Models, Logistic Regression, and Survival Analysis. New York, NY: Springer (2001). doi: 10.1007/978-1-4757-3462-1

[28] Eng C Dasari A Lonardi S Garcia-Carbonero R Elez E Yoshino T . Fruquintinib versus placebo in patients with refractory metastatic colorectal cancer: safety analysis of FRESCO-2. Oncologist. (2025) 30:oyae360. doi: 10.1093/oncolo/oyae360 40163688 PMC11957243

[29] Wang Y He Y Wang Z Zhong H Niu Z Yang S . Safety of fruquintinib in Chinese patients with colorectal cancer: an age subgroup analysis from a phase IV real-world clinical practice study. Ther Adv Med Oncol. (2025) 17:17588359251363537. doi: 10.1177/17588359251363537 40904832 PMC12402659

[30] National Cancer Institute. Common Terminology Criteria for Adverse Events (CTCAE) Version 5.0. Maryland: U.S. Department of Health and Human Services (2017).

[31] Williams PM Forbes T P LS Cole KD He H Karlovich C . Validation of ctDNA quality control materials through a precompetitive collaboration of the foundation for the national institutes of health. JCO Precis Oncol. (2021) 5:PO.20.00528. doi: 10.1200/PO.20.00528 34250423 PMC8232894

[32] Roazzi L Patelli G Bencardino KB Amatu A Bonazzina E Tosi F . Ongoing clinical trials and future research scenarios of circulating tumor DNA for the treatment of metastatic colorectal cancer. Clin Colorectal Cancer. (2024) 23:295–308. doi: 10.1016/j.clcc.2024.02.001 38519391

[33] Cremolini C Rossini D Dell'Aquila E Lonardi S Conca E Del Re M . Rechallenge for patients with RAS and BRAF wild-type metastatic colorectal cancer with acquired resistance to first-line cetuximab and irinotecan: a phase 2 single-arm clinical trial. JAMA Oncol. (2019) 5:343–50. doi: 10.1001/jamaoncol.2018.5080 30476968 PMC6439839

[34] Lockwood CM Borsu L Cankovic M Earle JSL Gocke CD Hameed M . Recommendations for cell-free DNA assay validations: a joint consensus recommendation of the Association for Molecular Pathology and College of American Pathologists. J Mol Diagn. (2023) 25:876–97. doi: 10.1016/j.jmoldx.2023.09.004 37806433

