# Supplementary material for: Early circulating tumor DNA dynamics predict fruquintinib efficacy in refractory metastatic colorectal cancer before imaging
Source: Front Med (Lausanne). 2026 Jun 12;13:1833355. doi: 10.3389/fmed.2026.1833355 (PMC13303014; doi:10.3389/fmed.2026.1833355)
Supplement: Supplementary file 1 [file Table_1.docx]

**Early Circulating Tumor DNA Dynamics Predict Fruquintinib Efficacy in Refractory Metastatic Colorectal Cancer Before Imaging**

**Authors:** Hebin Hou**^1^**,Pingping Liu**^1^**,Xiaohuan Dong**^1^**,Wei Gai**^1^**,Peng Jiang**^2^**,Fan Yang**^2^**,Chongli Hao**^2*^**

**Addresses:**

**^1^** Department of Gastroenterology, Tengzhou Central People's Hospital, Tengzhou 277500, Shandong, China.

**^2^** Department of Oncology, Tengzhou Central People's Hospital, Tengzhou 277500, Shandong, China.

**Correspondence: *** Chongli Hao**^2^**, **E-mail:** clhao@ldy.edu.rs

**Co-Author:** Hebin Hou**^1^**, E-mail: tzrmyyhhb@163.com

Pingping Liu**^1^**, E-mail: 13963298700@163.com

Xiaohuan Dong**^1^**, E-mail: dxh1004959540@163.com

Wei Gai**^1^**, E-mail: zzxhnkzk@126.com

Peng Jiang**^2^**, E-mail: jiangpeng20060114@163.com

Fan Yang**^2^**, E-mail: yf19831223@163.com

**
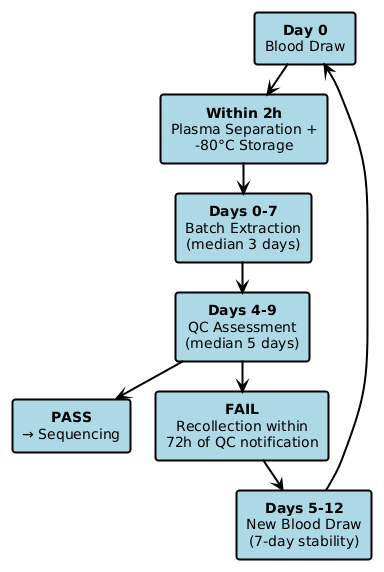
**

**Figure S1. Workflow Timeline for Blood Sample Processing, cfDNA Extraction, and Recollection.**Blood draw (Day 0) → plasma separation within 2 hours with immediate -80°C storage → batch cfDNA extraction (median 3 days) → QC assessment (median 5 days). Samples passing QC proceed to sequencing. Failed samples trigger recollection within 72 hours of QC notification, with new blood draw within manufacturer's 7-day stability window. QC = quality control; cfDNA = cell-free DNA.

**Table S2.** Comparison of Patients with Adequate vs. Insufficient Baseline ctDNA Samples.

| **Characteristic** | **Adequate Baseline ctDNA**  **(N = 114)** | **Insufficient Baseline ctDNA (N = 6)** | **P Value** |
| --- | --- | --- | --- |
| Age, years, median [IQR] | 59.3 [52.4–66.5] | 61.2 [54.1–67.8] | 0.48 |
| Male sex, n (%) | 72 (63.2) | 4 (66.7) | 0.86 |
| ECOG 2, n (%) | 18 (15.8) | 1 (16.7) | 0.95 |
| Liver metastasis, n (%) | 78 (68.4) | 4 (66.7) | 0.93 |
| Baseline SLD, mm, median [IQR] | 78.1 [48.9–122.1] | 82.3 [52.4–128.6] | 0.62 |
| Median PFS, months (95% CI) | 4.6 (3.9–5.3) | 4.1 (2.8–5.9) | 0.41 |

Note. No significant differences were observed between groups, suggesting exclusion of the 6 patients with insufficient baseline ctDNA did not introduce substantial selection bias.

**Table S3.** Comparison of Patients Included in vs. Excluded from Landmark Analysis.

| **Characteristic** | **Included in Landmark Analysis (N = 107)** | **Excluded from Landmark Analysis (N = 13)** | **P Value** |
| --- | --- | --- | --- |
| Age, years, median [IQR] | 59.2 [52.5–66.1] | 61.7 [54.3–68.2] | 0.34 |
| Sex, n (%) |  |  | 0.76 |
| Male | 66 (61.7) | 9 (69.2) |  |
| Female | 41 (38.3) | 4 (30.8) |  |
| ECOG performance status, n (%) |  |  | 0.08 |
| 0–1 | 93 (86.9) | 9 (69.2) |  |
| 2 | 14 (13.1) | 4 (30.8) |  |
| **Primary tumor site, n (%)** |  |  | 0.55 |
| Left-sided | 65 (60.7) | 7 (53.8) |  |
| Right-sided or transverse colon | 42 (39.3) | 6 (46.2) |  |
| Number of metastatic sites, n (%) |  |  | 0.21 |
| 1 | 43 (40.2) | 3 (23.1) |  |
| ≥2 | 64 (59.8) | 10 (76.9) |  |
| Liver metastasis, n (%) |  |  | 0.15 |
| Yes | 73 (68.2) | 11 (84.6) |  |
| No | 34 (31.8) | 2 (15.4) |  |
| Prior lines of systemic therapy, n (%) |  |  | 0.42 |
| 2 | 62 (57.9) | 6 (46.2) |  |
| ≥3 | 45 (42.1) | 7 (53.8) |  |
| Prior anti-VEGF exposure, n (%) |  |  | 0.38 |
| Yes | 65 (60.7) | 9 (69.2) |  |
| No | 42 (39.3) | 4 (30.8) |  |
| Baseline sum of longest diameters, mm, median [IQR] | 76.9 [48.1–118.4] | 94.2 [61.3–146.7] | 0.047 |
| Baseline CEA, ng/mL, median [IQR] | 58.7 [6.3–411.2] | 89.3 [12.4–502.6] | 0.21 |
| Baseline maximum VAF, %, median [IQR] | 1.81 [0.39–6.75] | 3.14 [0.86–9.42] | 0.043 |
| RAS/BRAF mutation status, n (%) |  |  | 0.56 |
| Mutant | 60 (56.1) | 8 (61.5) |  |
| Wild-type | 47 (43.9) | 5 (38.5) |  |
| Reason for exclusion, n (%) |  |  |  |
| Early progression before day 28 | — | 5 (38.5) |  |
| Death before day 28 | — | 3 (23.1) |  |
| Sample quality control failure | — | 3 (23.1) |  |
| Withdrawal of consent | — | 2 (15.4) |  |

Note. ECOG = Eastern Cooperative Oncology Group; VEGF = vascular endothelial growth factor; CEA = carcinoembryonic antigen; VAF = variant allele frequency; IQR = interquartile range. P values were calculated using Wilcoxon rank-sum test for continuous variables and chi-square test or Fisher's exact test for categorical variables, as appropriate. Boldface indicates statistical significance (P < 0.05). Excluded patients had significantly higher baseline tumor burden and higher baseline maximum VAF, consistent with their early progression or death before the day-28 landmark.

**Table S4.** Circulating Tumor DNA Sequencing Quality Control Metrics.

| **Metric** | **Value, Median [IQR]** | **Prespecified Threshold** | **Samples Passing, n (%)** |
| --- | --- | --- | --- |
| Deduplicated effective depth (×) | 7,512 [6,123–9,026] | ≥5,000× | 337 (99.1) |
| Q30 proportion (%) | 91.8 [88.4–94.6] | ≥85.0% | 338 (99.4) |
| UMI collapsing rate (%) | 82.3 [74.3–88.6] | No threshold | — |
| Coverage uniformity (CV) | 0.18 [0.14–0.21] | ≤0.25 | 336 (98.8) |
| Background mutation rate (per Mb) | 0.42 [0.27–0.63] | ≤1.00 | 339 (99.7) |
| cfDNA yield (ng) | 12.7 [7.9–21.4] | ≥2.0 | 338 (99.4) |
| Overall sample pass rate | — | — | 334 (98.2) |
| Additional Quality Metrics | Value |  |  |
| Total samples sequenced, N | 340 |  |  |
| Baseline samples, n | 120 |  |  |
| Day 14 samples, n | 113 |  |  |
| Day 28 samples, n | 107 |  |  |
| Samples requiring recollection, n (%) | 6 (1.8) |  |  |
| Patients with CHIP at baseline, n (%) | 17 (14.2) |  |  |
| CHIP loci filtered, total N | 43 |  |  |

Note. IQR = interquartile range; UMI = unique molecular identifier; CV = coefficient of variation; cfDNA = cell-free DNA; Mb = million bases; CHIP = clonal hematopoiesis of indeterminate potential. Values are based on 340 sequenced samples from 120 patients. Six samples (1.8%) initially failed quality control and were successfully recollected within 72 hours. All passing samples met the prespecified quality thresholds. CHIP variants were identified through paired white blood cell sequencing and excluded from circulating tumor DNA metric calculations.

**Table S5.** Baseline Characteristics According to Day-28 Circulating Tumor DNA Dynamic Strata

| **Characteristic** | **Clearance**  **(N = 19)** | **Decrease (N = 47)** | **Stable (N = 25)** | **Increase (N = 16)** | **P Value** |
| --- | --- | --- | --- | --- | --- |
| Age, years, median [IQR] | 58.4 [51.9–64.7] | 59.7 [53.1–66.8] | 60.2 [52.8–67.3] | 58.9 [51.4–65.2] | 0.62 |
| **Sex, n (%)** |  |  |  |  | 0.48 |
| Male | 12 (63.2) | 29 (61.7) | 15 (60.0) | 10 (62.5) |  |
| Female | 7 (36.8) | 18 (38.3) | 10 (40.0) | 6 (37.5) |  |
| ECOG performance status, n (%) |  |  |  |  | 0.31 |
| 0–1 | 18 (94.7) | 41 (87.2) | 21 (84.0) | 13 (81.3) |  |
| 2 | 1 (5.3) | 6 (12.8) | 4 (16.0) | 3 (18.7) |  |
| **Primary tumor site, n (%)** |  |  |  |  | 0.57 |
| Left-sided | 12 (63.2) | 29 (61.7) | 15 (60.0) | 9 (56.3) |  |
| Right-sided or transverse colon | 7 (36.8) | 18 (38.3) | 10 (40.0) | 7 (43.7) |  |
| Number of metastatic sites, n (%) |  |  |  |  | 0.19 |
| 1 | 9 (47.4) | 20 (42.6) | 9 (36.0) | 5 (31.3) |  |
| ≥2 | 10 (52.6) | 27 (57.4) | 16 (64.0) | 11 (68.7) |  |
| Liver metastasis, n (%) |  |  |  |  | 0.08 |
| Yes | 11 (57.9) | 31 (66.0) | 18 (72.0) | 13 (81.3) |  |
| No | 8 (42.1) | 16 (34.0) | 7 (28.0) | 3 (18.7) |  |
| Prior lines of systemic therapy, n (%) |  |  |  |  | 0.44 |
| 2 | 12 (63.2) | 28 (59.6) | 14 (56.0) | 8 (50.0) |  |
| ≥3 | 7 (36.8) | 19 (40.4) | 11 (44.0) | 8 (50.0) |  |
| **Prior anti-VEGF exposure, n (%)** |  |  |  |  | 0.52 |
| Yes | 11 (57.9) | 28 (59.6) | 16 (64.0) | 10 (62.5) |  |
| No | 8 (42.1) | 19 (40.4) | 9 (36.0) | 6 (37.5) |  |
| Baseline SLD, mm, median [IQR] | 68.4 [41.2–94.7] | 74.3 [46.8–112.5] | 81.6 [51.3–124.8] | 89.7 [58.4–136.2] | 0.04 |
| Baseline CEA, ng/mL, median [IQR] | 42.6 [5.1–312.8] | 54.3 [6.2–389.4] | 67.8 [7.4–446.2] | 81.4 [8.9–498.7] | 0.12 |
| Baseline maximum VAF, %, median [IQR] | 1.12 [0.28–4.36] | 1.64 [0.37–5.82] | 2.18 [0.46–7.43] | 2.34 [0.51–8.67] | 0.03 |
| **RAS/BRAF mutation status, n (%)** |  |  |  |  | 0.38 |
| Mutant | 10 (52.6) | 26 (55.3) | 15 (60.0) | 9 (56.3) |  |
| Wild-type | 9 (47.4) | 21 (44.7) | 10 (40.0) | 7 (43.7) |  |

Note. ECOG = Eastern Cooperative Oncology Group; VEGF = vascular endothelial growth factor; SLD = sum of longest diameters; CEA = carcinoembryonic antigen; VAF = variant allele frequency; IQR = interquartile range. P values were calculated using Kruskal–Wallis test for continuous variables and chi-square test for categorical variables. Boldface indicates statistical significance (P < 0.05). Patients with clearance had numerically lower baseline tumor burden and lower baseline VAF, while those with increase had the highest baseline values.

**Table S6.** Concordance Between Day-14 and Day-28 ctDNA Classifications.

| **Day-14 Classification** | **Day-28 Clearance** | **Day-28 Decrease** | **Day-28 Stable** | **Day-28 Increase** | **Total** |
| --- | --- | --- | --- | --- | --- |
| Clearance | 6 (75.0) | 2 (25.0) | 0 (0) | 0 (0) | 8 |
| Decrease | 10 (32.3) | 18 (58.1) | 3 (9.7) | 0 (0) | 31 |
| Stable | 3 (7.1) | 22 (52.4) | 14 (33.3) | 3 (7.1) | 42 |
| Increase | 0 (0) | 5 (19.2) | 8 (30.8) | 13 (50.0) | 26 |
| Total | 19 | 47 | 25 | 16 | 107 |

Note. Data are presented as number of patients (row percentage in parentheses). Overall concordance = 47.7% (51/107). Most discordant cases (52.3%) showed improvement from day-14 to day-28, indicating continued molecular response beyond the first two weeks of treatment and supporting day-28 as the optimal early landmark.

**Table S7.** Association Between Day-28 ctDNA Dynamics and Objective Response

| **ctDNA Stratum** | **N** | **CR/PR, n (%)** | **SD/PD, n (%)** |
| --- | --- | --- | --- |
| Clearance | 19 | 7 (36.8) | 12 (63.2) |
| Decrease | 47 | 6 (12.8) | 41 (87.2) |
| Stable | 25 | 1 (4.0)* | 24 (96.0) |
| Increase | 16 | 1 (6.3)* | 15 (93.7) |
| Stable/Increase (combined) | 41 | 2 (4.9) | 39 (95.1) |
| Total | 107 | 15 (14.0) | 92 (86.0) |

Note. CR = complete response; PR = partial response; SD = stable disease; PD = progressive disease. Data are derived from Section 3.4 of the main manuscript. *The two objective responses in the stable/increase group occurred in stable (n = 1) and increase (n = 1) strata, but exact distribution within the combined group is not available from the reported data. Chi-square test for association between ctDNA strata (clearance/decrease vs. stable/increase) and objective response: P < 0.001.

**Table S8.** Multivariable Cox Regression Analysis for Overall Survival.

| **Variable** | **Adjusted Hazard Ratio (95% CI)** | **P Value** |
| --- | --- | --- |
| ctDNA dynamics (vs. stable/increase) |  |  |
| Clearance | 0.44 (0.27–0.72) | 0.001 |
| Decrease | 0.67 (0.45–0.99) | 0.046 |
| Age ≥65 years (vs. <65) | 1.21 (0.84–1.74) | 0.31 |
| Male sex (vs. female) | 0.94 (0.66–1.34) | 0.73 |
| ECOG 2 (vs. 0–1) | 1.76 (1.16–2.67) | 0.008 |
| Right-sided primary (vs. left) | 1.31 (0.92–1.87) | 0.13 |
| Liver metastasis (vs. none) | 1.42 (0.97–2.08) | 0.07 |
| ≥2 metastatic sites (vs. 1) | 1.48 (1.01–2.17) | 0.045 |
| Prior anti-VEGF exposure (vs. none) | 1.23 (0.86–1.76) | 0.26 |
| Baseline SLD (per 10-mm increase) | 1.09 (1.02–1.17) | 0.012 |
| Baseline maximum VAF (per 1% increase) | 1.06 (1.01–1.12) | 0.028 |

Note. ctDNA = circulating tumor DNA; CI = confidence interval; ECOG = Eastern Cooperative Oncology Group; VEGF = vascular endothelial growth factor; SLD = sum of longest diameters; VAF = variant allele frequency. Model adjusted for all nine prespecified covariates. Stable and increase groups were combined as reference due to similar hazard ratios in univariable analysis (stable vs. increase: HR, 1.31; 95% CI, 0.76–2.26; P = 0.33). Boldface indicates statistical significance (P < 0.05). The proportional hazards assumption was satisfied for all covariates (global Schoenfeld test, P = 0.31).

**Table S9.** Sensitivity Analyses for the Association Between Circulating Tumor DNA Dynamics and Progression-Free Survival.

| **Sensitivity Analysis** | **N** | **Adjusted Hazard Ratio (95% CI)** | **P Value** |
| --- | --- | --- | --- |
| Primary analysis (clearance/decrease vs. stable/increase) | 107 | 0.54 (0.38–0.77) | <0.001 |
| Alternative threshold: 30% decrease (decrease redefined as ≥30% reduction) | 107 | 0.57 (0.39–0.80) | 0.002 |
| Alternative threshold: 70% decrease (decrease redefined as ≥70% reduction) | 107 | 0.49 (0.33–0.73) | 0.001 |
| Using ctDNA burden instead of maximum VAF (sum of all non-synonymous mutation VAFs) | 107 | 0.61 (0.44–0.86) | 0.004 |
| Time-varying covariate model (including patients with events before day 28) | 111 | 0.58 (0.41–0.84) | 0.003 |
| Complete case analysis (no imputation for missing covariates) | 102 | 0.55 (0.38–0.79) | 0.001 |
| Excluding patients with baseline ctDNA undetectable (n = 12 with baseline VAF = 0) | 95 | 0.52 (0.36–0.75) | <0.001 |
| **Model Performance Across Sensitivity Analyses** | | **Harrell's C-Index (95% CI)** | |
| Primary model (clinical + ctDNA dynamics) | | 0.71 (0.66–0.77) | |
| 30% threshold model | | 0.70 (0.65–0.76) | |
| 70% threshold model | | 0.69 (0.64–0.75) | |
| ctDNA burden model | | 0.68 (0.63–0.74) | |
| Time-varying covariate model | | 0.69 (0.64–0.75) | |
| **Sensitivity Analysis Parameter** | **Clearance/Decrease, n (%)** | **Stable/Increase, n (%)** | **Events, n** |
| Primary analysis | 66/107 (61.7) | 41/107 (38.3) | 89 |
| 30% threshold | 71/107 (66.4) | 36/107 (33.6) | 89 |
| 70% threshold | 52/107 (48.6) | 55/107 (51.4) | 89 |
| ctDNA burden metric | 64/107 (59.8) | 43/107 (40.2) | 89 |
| Time-varying model | 68/111 (61.3) | 43/111 (38.7) | 97 |
| Complete case analysis | 63/102 (61.8) | 39/102 (38.2) | 85 |
| Excluding baseline undetectable | 66/95 (69.5) | 29/95 (30.5) | 82 |

Note. ctDNA = circulating tumor DNA; CI = confidence interval; VAF = variant allele frequency. All models adjusted for the nine prespecified covariates (age, sex, ECOG performance status, primary tumor laterality, liver metastasis, number of metastatic sites, prior anti-VEGF exposure, baseline sum of longest diameters, and baseline maximum VAF, except where VAF was replaced by ctDNA burden). For threshold analyses, hazard ratios compare combined clearance/decrease with stable/increase. The time-varying covariate model includes 8 patients who had events before day 28 and were excluded from the landmark analysis. Boldface indicates statistical significance (P < 0.05). All sensitivity analyses yielded effect estimates consistent with the primary analysis, demonstrating robustness of the findings.

**Table S10.** Post-Progression Anticancer Treatments.

| **Treatment Category** | | **Patients Receiving Treatment, n (%)** | | **Time to Initiation, months, median [IQR]** | |
| --- | --- | --- | --- | --- | --- |
| Any subsequent therapy | | 91 (75.8) | | 5.2 [3.8–7.4] | |
| By treatment type | |  | |  | |
| Chemotherapy (FOLFOX/FOLFIRI or other) | | 49 (40.8) | | 5.6 [4.3–7.9] | |
| Anti-VEGF agents (bevacizumab, aflibercept) | | 37 (30.8) | | 5.1 [3.9–7.0] | |
| Regorafenib | | 28 (23.3) | | 4.9 [3.7–6.6] | |
| Anti-EGFR antibodies (cetuximab, panitumumab) | | 23 (19.2) | | 6.3 [5.1–8.4] | |
| Clinical trial participation | | 16 (13.3) | | 6.8 [5.3–8.9] | |
| Immunotherapy (anti-PD-1/PD-L1) | | 7 (5.8) | | 7.7 [6.2–10.1] | |
| Best supportive care only | | 29 (24.2) | | 3.9 [2.4–6.1] | |
| **Post-Progression Treatment by ctDNA Stratum** | **Clearance**  **(N = 19)** | **Decrease**  **(N = 47)** | **Stable**  **(N = 25)** | **Increase**  **(N = 16)** | **P Value** |
| Received any subsequent therapy, n (%) | 16 (84.2) | 37 (78.7) | 18 (72.0) | 10 (62.5) | 0.19 |
| Time to subsequent therapy, months, median [IQR] | 6.8 [5.2–8.9] | 5.4 [4.1–7.6] | 4.7 [3.4–6.3] | 3.8 [2.6–5.1] | 0.008 |
| Received regorafenib, n (%) | 6 (31.6) | 13 (27.7) | 6 (24.0) | 3 (18.8) | 0.62 |
| Received anti-VEGF agents, n (%) | 7 (36.8) | 16 (34.0) | 8 (32.0) | 4 (25.0) | 0.71 |
| **Reason for No Subsequent Therapy** | | **n (N = 29)** | | **% of 120** | |
| Rapid clinical deterioration | | 14 | | 48.3 | |
| Patient preference | | 7 | | 24.1 | |
| Physician decision (no further options) | | 5 | | 17.2 | |
| Lost to follow-up | |  | | 10.3 | |

Note. VEGF = vascular endothelial growth factor; EGFR = epidermal growth factor receptor; IQR = interquartile range; ctDNA = circulating tumor DNA. Percentages for treatment categories are based on the full enrolled cohort (N = 120). Patients may have received multiple lines or classes of subsequent therapy; each category is counted separately. Time to initiation is defined as months from first fruquintinib dose to start of the specified subsequent therapy. Boldface indicates statistical significance (P < 0.05) for comparison across ctDNA strata using Kruskal–Wallis test.
